# Efficacy and Safety of Amrubicin in Small Cell Carcinoma Previously Treated with Immune Checkpoint Inhibitors and Chemotherapy

**DOI:** 10.3390/cancers14163953

**Published:** 2022-08-16

**Authors:** Tadashi Nishimura, Hajime Fujimoto, Takumi Fujiwara, Kentaro Ito, Atsushi Fujiwara, Hisamichi Yuda, Hidetoshi Itani, Masahiro Naito, Shuji Kodama, Akihiko Yagi, Valeria Fridman D’Alessandro, Taro Yasuma, Kazuki Furuhashi, Haruko Saiki, Tomohito Okano, Atsushi Tomaru, Motoaki Tanigawa, Corina N. D’Alessandro-Gabazza, Esteban C. Gabazza, Masamichi Yoshida, Osamu Hataji, Hidenori Ibata, Tetsu Kobayashi

**Affiliations:** 1 Department of Pulmonary Medicine, Mie Chuo Medical Center, Tsu 514-1101, Mie, Japan; 2 Department of Pulmonary and Critical Care Medicine, Mie University Faculty and Graduate School of Medicine, Tsu 514-8507, Mie, Japan; 3 Department of Genomic Medicine, Mie University Hospital, Tsu 514-8507, Mie, Japan; 4 Respiratory Center, Matsusaka Municipal Hospital, Matsusaka 515-8544, Mie, Japan; 5 Department of Pulmonary Medicine, Mie Prefectural General Medical Center, Yokkaichi 510-8561, Mie, Japan; 6 Department of Pulmonary Medicine, Kuwana City Medical Center, Kuwana 511-0061, Mie, Japan; 7 Department of Respiratory Medicine, Ise Red Cross Hospital, Ise 516-8512, Mie, Japan; 8 Department of Immunology, Mie University Faculty and Graduate School of Medicine, Tsu 514-8507, Mie, Japan

**Keywords:** immune checkpoint inhibitor, small cell lung cancer, amrubicin

## Abstract

**Simple Summary:**

Therapeutic efficacy of chemotherapy combined with immune checkpoint inhibitors as first-line therapy has been previously demonstrated in extensive-stage small cell lung cancer. However, there are no reports of any cytotoxic drug that is effective as second-line therapy in extensive-stage small cell lung cancer patients previously treated with chemotherapy and immune checkpoint inhibitors as a first-line treatment. In the present study, we retrospectively evaluated patients with extensive-stage small cell lung cancer to clarify whether the previous treatment with chemotherapy and immune checkpoint inhibitors impacts the efficacy and safety of amrubicin as a second-line treatment. This study shows that the efficacy and safety of amrubicin in extensive-stage small cell lung cancer remains unchanged irrespective of previous treatment with chemotherapy and immune checkpoint inhibitors.

**Abstract:**

Adding an immune checkpoint inhibitor to chemotherapy to treat extensive-stage small cell lung cancer is effective. However, there are no reports of an effective second-line treatment in patients previously treated with chemotherapy and immune checkpoint inhibitors as a first-line treatment. Here, we assessed the efficacy and safety of amrubicin as a second-line treatment for extensive-stage small cell lung cancer after chemotherapy and immune checkpoint inhibitor combination therapy. The study enrolled 150 patients with extensive-stage small cell lung cancer. The efficacy and the incidence of adverse events were compared between patients previously treated with immune checkpoint inhibitors and patients without previous immune checkpoint inhibitor treatment. One hundred and twenty-three patients were eligible. There was no difference in objective response rate, time-to-treatment failure, progression-free survival, and overall survival between both groups. The incidence of adverse events was similar in both treatment groups. Pretreatment with immune checkpoint inhibitors was not associated with an increase in amrubicin-related adverse events. This study shows that the efficacy of amrubicin in extensive-stage small cell lung cancer remains unchanged irrespective of previous treatment with immune checkpoint inhibitors. Amrubicin-related adverse events did not increase in patients previously treated with immune checkpoint inhibitors.

## 1. Introduction

Small cell lung cancer (SCLC) accounts for about 15% of all lung cancers. More than half of such patients are diagnosed with extensive-stage small cell lung cancer (ES-SCLC) [1]. The addition of immune checkpoint inhibitors (ICIs) to chemotherapy for treating ES-SCLC is effective and is currently recommended as the first-line treatment [2,3,4,5,6,7]. However, there are no reports of any effective second-line therapeutic protocol for treating ES-SCLC in patients previously treated with chemotherapy and ICI combination therapy. On the other hand, amrubicin, a topoisomerase inhibitor, is effective as a second-line treatment in sensitive and refractory SCLC [8,9,10]. Therefore, the Japanese Cancer Guidelines recommend amrubicin as second-line therapy for SCLC in Japan. In this study, we analyzed the efficacy and safety of amrubicin as a second-line treatment for ES-SCLC in patients previously treated with ICIs and chemotherapy.

## 2. Materials and Methods

### 2.1. Patients and Study Design

**Figure 1:**
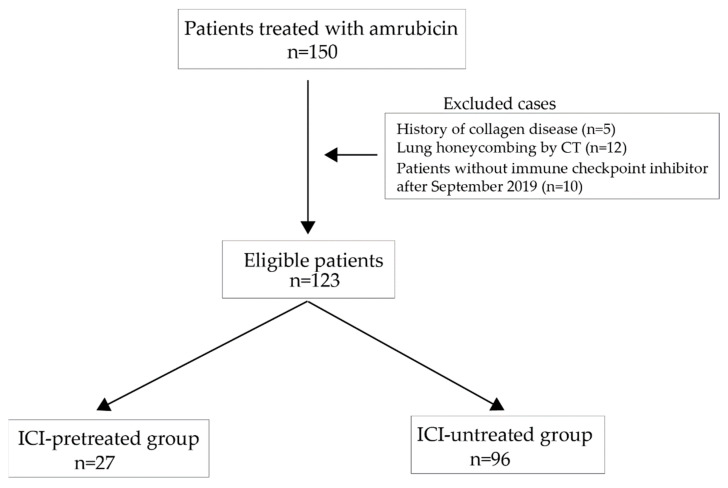
Study flow chart. The patients were divided into immune checkpoint inhibitor (ICI)-treated and ICI-untreated groups.

We evaluated 150 patients who received amrubicin as a second-line treatment from April 2012 through to December 2021 in six different institutions in Japan (Figure 1). Amrubicin was administered at a dose rate of 40 or 35 mg/m^2^ for 3 days every 3 weeks. Amrubicin may exacerbate interstitial disease in patients with previous history of interstitial pneumonia. ICIs may also induce interstitial pneumonia. This study included patients that have used amrubicin before the availability of ICIs. Therefore, to avoid bias in the backgrounds of the treatment groups, in the present study, patients with interstitial pneumonia and/or with collagen disease, in whom the use of ICIs is contraindicated, were excluded from the study groups. There were 123 eligible patients. Eligible patients were categorized into 2 groups: previously treated (ICI-pretreated group) and not previously treated with ICIs (ICI-untreated group). The overall response rate, disease control rate, time-to-treatment failure, progression-free survival, and overall survival were assessed and compared between groups.

### 2.2. Ethical Statement

The Committee for Clinical Investigation of Mie Chuo Medical Center approved the protocol of the current clinical investigation (Approval No MCERB-202144; approval date: 11 January 2022). In addition, the Ethical Review Board of the remaining five participating institutions also approved the protocol of the clinical investigation (Approval No 2021-S12, H2022-036, J-162-220121-12-1, ER2021-102, 204).

### 2.3. Statistical Analysis

The Response Evaluation Criteria in Solid Tumors (RECIST) version 1.1 was used to determine the overall response rate and disease control rate. The progression-free survival, time-to-treatment failure, and overall survival were assessed using the Kaplan–Meier curve and log-rank test. Categorical variables were evaluated using Fisher’s test and multivariate analysis was performed using the Cox proportional hazards regression. The hazard ratios were also calculated after adjusting for confounding factors, including age, gender, Eastern Cooperative Oncology Group performance status (ECOG PS), sensitivity to previous chemotherapy, and the presence of liver or adrenal metastasis. The inverse probability of treatment weighting (IPTW) was evaluated using a propensity score. The Common Terminology Criteria for Adverse Events (CTCAEs) version 5.0 was used to evaluate adverse events. A *p* < 0.05 was considered significant. The statistical analysis was performed using the R software package version 4.0.3 (R Development Core Team, Vienna, Austria) and the EZR version 1.54 (Saitama Medical Center, Jichi Medical University, Saitama, Japan) [11].

## 3. Results

### 3.1. Patients’ Characteristics

The study included 150 patients, and 123 of them were eligible. There were 27 patients in the ICI-pretreated group and 96 in the ICI-untreated group (Figure 1). The characteristics of the patients from these groups are shown in Table 1.

### 3.2. No Difference in Tumor Response between ICI-Pretreated and ICI-Untreated Groups

The overall response rate was 30.8% in the ICI-pretreated group and 22.5% in the ICI-untreated group. The disease control rate was 61.5% in the ICI-pretreated group and 67.4% in the ICI-untreated group. There was no significant statistical difference between groups (Table 2).

### 3.3. No Difference in Time-to-Treatement Failure, Progression-Free Survival, and Overall Survival between ICI-Pretreated and ICI-Untreated Groups

**Figure 2:**
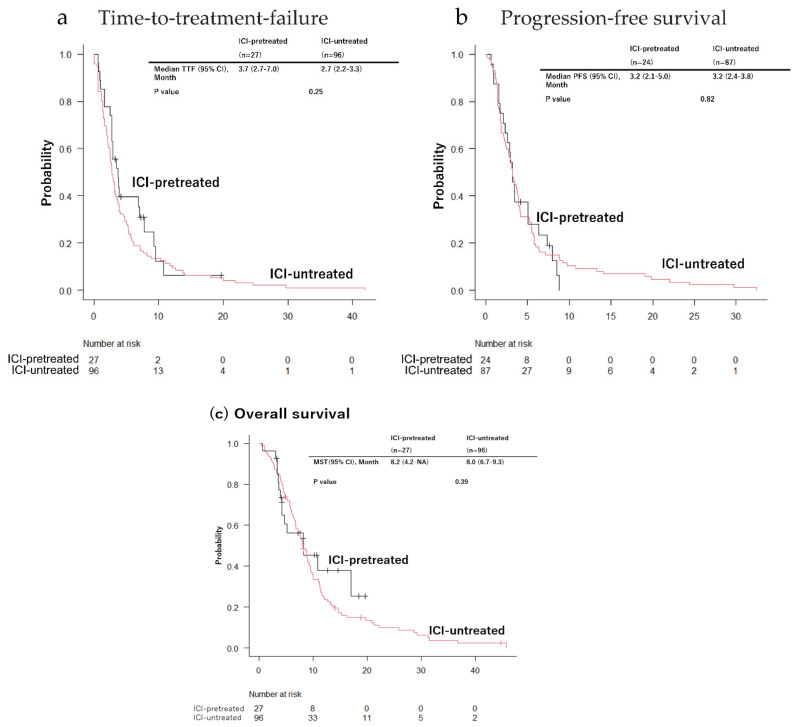
Kaplan–Meier curves of (**a**) time-to-treatment failure, (**b**) progression-free survival, and (**c**) overall survival. CI: confidence interval; ICI: immune checkpoint inhibitor; MST: median survival time; NA: not assessed; PFS: progression-free survival; TTF: time-to-treatment failure.

The time-to-treatment failure (3.7 vs. 2.7 months), the median progression-free survival (3.2 vs. 3.2 months), and the overall survival (8.2 vs. 8.0 months) were not significantly different between the ICI-pretreated and ICI-untreated groups (Figure 2a–c).

### 3.4. Performance Status, Adrenal Metastasis, and Pleural Effusion Predicted a Poor Prognosis

Following univariate analysis, performance status, liver metastasis, and adrenal metastasis were found to be significantly correlated with the time-to-treatment failure, median progression-free survival, and overall survival. Malignant pleural effusion was significantly correlated with overall survival (Table 3). Following multivariate analysis, performance status and adrenal metastasis were found to be independent predicting factors for time-to-treatment failure, median progression-free survival, and overall survival. Age, performance status, malignant pleural effusion, and adrenal metastasis were significant predicting factors for overall survival (Table 3). The hazard ratios for the time-to-treatment failure, progression-free survival, and overall survival after adjustment to the propensity score matching were not significantly different between ICI-pretreated and ICI-untreated groups, suggesting that the use of ICIs did not affect the response to amrubicin (Table 4).

### 3.5. Pretreatment with ICIs Exerted No Influence on Adverse Events

Hematologic adverse events and pneumonitis were investigated and compared between ICI-pretreated and ICI-untreated groups (Table 5). No significant difference in the incidence of febrile neutropenia (22.2% vs. 24%), grade 3 adverse events, or pneumonitis (3.7% vs. 4.2%) was observed between the ICI-pretreated and ICI-untreated groups. One patient (4.3%) from the ICI-pretreated group and 11 (11.5%) from the ICI-untreated group discontinued amrubicin due to adverse events.

## 4. Discussion

The results of this study showed that pretreatment with ICIs exerts no effect on the efficacy of amrubicin in ES-SCLC. To the best of our knowledge, this is the first study showing the efficacy of amrubicin in ES-SCLC patients previously treated with ICIs.

Previous studies have shown a poor efficacy of ICI monotherapy as a second-line treatment in SCLC. Short progression-free survival of 1.4 to 1.9 months has been reported [12,13,14]. By contrast, a progression-free survival of 4.0 months was reported in a phase II trial where pembrolizumab was used in addition to amrubicin as a second-line treatment for ES-SCLC [15]. Anthracyclines such as amrubicin promote the mobilization and recruitment of T-cell antigen-presenting cells to the tumor sites, apart from stimulating immunogenic cell death and interferon secretion [16,17,18]. Therefore, amrubicin is expected to be effective in combination with immunotherapy [8,13]. On this basis, we recommend using immunotherapy in combination with chemotherapy to treat SCLC patients.

Several studies have previously assessed whether pretreatment with ICIs improves the efficacy of subsequent therapy for non-small cell lung cancer [19,20,21,22,23,24]. In some cases, pretreatment with ICIs improved the overall response rate, but not progression-free survival or overall survival [22,24]. Residual effects of ICIs for 2 to 6 months after their initial administration have been reported [25,26,27]. In the present study, we have not assessed the residual effects of ICIs, based on the results of previous reports.

We speculate that the residual effects of ICI are not so long-lasting. Therefore, we believe that patients with ES-SCLC should be treated with first-line chemotherapy and ICI combination therapy for as long as possible to achieve long-term survival.

Previous trials have shown that chemotherapy and ICI combination therapy have not induced any unpredictable adverse events, although adverse events resulting from ICI and chemotherapy treatment were observed [4,7,15]. In a phase II trial using pembrolizumab in addition to amrubicin as a second-line treatment, no unusual adverse events were observed, and the frequency of hematologic adverse events was similar to that observed in our current study [15]. In addition, we found a lower frequency of pneumonitis compared to previous studies where the combination of ICIs and amrubicin was indicated. It is possible that the concurrent use of ICIs and amrubicin increases the frequency of pneumonitis and that the inducibility of pneumonitis differs between PD-1 and PD-L1 antibodies. In brief, the results of our current study indicate that adverse events caused by amrubicin are not enhanced by previous treatment with ICIs.

Limitations of our study include the retrospective nature of the study, the small number of patients previously treated with ICIs, and the short follow-up period. Further limitations are potential bias relating to the patients’ backgrounds, as only patients who have shown a variable or refractory response to previous treatment were found to be eligible for a second-line treatment, and failure to measure the residual blood levels of ICIs. In addition, it is currently unknown whether the efficacy of ICIs in SCLC is related to PD-L1 expression or tumor mutation burden, and no predictive biomarkers have been identified [12,14]. However, genetic analysis has recently identified subgroups with different responsiveness to chemotherapy and ICI combination therapy [28,29]. Prospective clinical trials, including a large population of patients with matched subgroups in terms of response to treatment, should be conducted in future studies to overcome these limitations.

## 5. Conclusions

This study shows that the efficacy of amrubicin in ES-SCLC remains unchanged irrespective of previous treatment with ICIs. In addition, no increase in adverse events was observed in cases pretreated with ICIs.

## Figures and Tables

**Table 1:** Patients’ characteristics.

Factor	Group	ICI- Pretreated	ICI- Untreated	*p*- Value
n		27	96	
Gender	Male	26 (96.3)	79 (82.3)	0.119
	Female	1 (3.7)	17 (17.7)	
Age (%)	<70	10 (37.0)	48 (50.0)	0.279
	≥70	17 (63.0)	48 (50.0)	
ECOG performance status (%)	0	8 (29.6)	34 (35.4)	0.941
	1	16 (59.3)	49 (51.0)	
	2	3 (11.1)	11 (11.5)	
	3	0 (0.0)	2 (2.1)	
Smoking status (%)	Yes	27 (100.0)	96 (100.0)	NA
Previous treatment (%)	CBDCA+etoposide	0 (0.0)	82 (85.4)	<0.001
	CBDCA+etoposide+atezolizumab	23 (85.2)	0 (0.0)	
	CBDCA+etoposide+durvalumab	4 (14.8)	0 (0.0)	
	CBDCA+irinotecan	0 (0.0)	4 (4.2)	
	CDDP+etoposide	0 (0.0)	7 (7.3)	
	CDDP+irinotecan	0 (0.0)	3 (3.1)	
Sensitivity of previous chemotherapy (%)	Sensitive (>90 days of last chemotherapy)	6 (22.2)	46 (47.9)	0.026
	Refractory (relapsed ≤90 days of last chemotherapy)	21 (77.8)	50 (52.1)	
Brain metastasis (%)	Negative	19 (70.4)	60 (62.5)	0.503
	Positive	8 (29.6)	36 (37.5)	
Liver metastasis (%)	Negative	21 (77.8)	62 (64.6)	0.248
	Positive	6 (22.2)	34 (35.4)	
Malignant pleural effusion (%)	Negative	20 (74.1)	72 (75.0)	1
	Positive	7 (25.9)	24 (25.0)	
Bone metastasis (%)	Negative	16 (59.3)	67 (69.8)	0.354
	Positive	11 (40.7)	29 (30.2)	
Adrenal metastasis (%)	Negative	19 (70.4)	78 (81.2)	0.285
	Positive	8 (29.6)	18 (18.8)	
Discontinuation of amrubicin due to adverse effects	Negative	22 (95.7)	85 (88.5)	0.457
	Positive	1 (4.3)	11 (11.5)	

CBDCA: carboplatin; CDDP: cisplatin; ECOG: Eastern Cooperative Oncology Group; NA: not assessed.

**Table 2:** Tumor response rate and disease control rate.

	ICI-Pretreated	ICI-Untreated	*p*-Value
n	27	96	
Complete response (%)	0 (0.0)	3 (3.1)	0.51
Partial response (%)	8 (29.6)	17 (17.7)	
Stable disease (%)	8 (29.6)	40 (41.7)	
Progressive disease (%)	10 (37.0)	29 (30.2)	
Not evaluated (%)	1 (3.7)	7 (7.3)	
Overall response rate (%)	8 (30.8, 95% CI 14.3–51.8)	20 (22.5, 95% CI 14.3–32.6)	
Disease control rate (%)	16 (61.5, 95% CI 40.6–79.8)	60 (67.4, 95% CI 56.7–77.0)	

CI: confidence interval; ICI: immune checkpoint inhibitor.

**Table 3:** Univariate and multivariate analysis.

		**Time-to-Treatment Failure**			
		**Univariate Analysis**		**Multivariate Analysis**	
**Factors**		**Hazard Ratio**	***p*** **Value**	**Hazard Ratio**	***p*** **Value**
Age	<70	Referent	0.57	Referent	0.53
	>=70	1.11 (0.77–1.6)		1.13 (0.77–1.68)	
Gender	Male	Referent	0.41	Referent	0.53
	Female	0.81 (0.49–1.34)		0.84 (0.48–1.46)	
Performance status *	0–2	Referent	0.00059	Referent	0.00092
	3	13.62 (3.07–60.4)		13.86 (2.93–65.62)	
Brain metastasis	Negative	Referent	0.21	Referent	0.33
	Positive	1.28 (0.87–1.87)		1.24 (0.81–1.89)	
Liver metastasis	Negative	Referent	0.0097	Referent	0.18
	Positive	1.69 (1.14–2.53)		1.36 (0.87–2.14)	
M. pleural effusion	Negative	Referent	0.12	Referent	0.23
	Positive	1.41 (0.92–2.15)		1.32 (0.84–2.07)	
Bone metastasis	Negative	Referent	0.41	Referent	0.48
	Positive	1.18 (0.8–1.74)		1.17 (0.76–1.79)	
Adrenal metastasis	Negative	Referent	0.007	Referent	0.027
	Positive	1.87 (1.19–2.96)		1.83 (1.07–3.11)	
Previous treatment	ICI-pretreated	Referent	0.26	Referent	0.11
	ICI-untreated	1.14 (0.9–1.44)		1.22 (0.95–1.57)	
		**Progression-Free Survival**			
		**Univariate Analysis**		**Multivariate Analysis**	
**Factor**		**Hazard Ratio**	***p*** **Value**	**Hazard Ratio**	***p*** **Value**
Age	<70	Referent	0.73	Referent	0.86
	>=70	0.93 (0.64–1.37)		0.97 (0.65–1.44)	
Gender	Male	Referent	0.94	Referent	0.62
	Female	0.98 (0.57–1.67)		1.16 (0.64–2.12)	
Performance status *	0–2	Referent	0.0051	Referent	0.0078
	3	21.53 (2.52–184.3)		20.72 (2.22–193.30)	
Brain metastasis	Negative	Referent	0.37	Referent	0.94
	Positive	1.20 (0.81–1.79)		0.98 (0.63–1.53)	
Liver metastasis	Negative	Referent	0.011	Referent	0.069
	Positive	1.74 (1.14–2.67)		1.58 (0.97–2.60)	
M. pleural effusion	Negative	Referent	0.19	Referent	0.47
	Positive	1.35 (0.86–2.1)		1.19 (0.74–1.91)	
Bone metastasis	Negative	Referent	0.51	Referent	0.96
	Positive	1.15 (0.76–1.75)		0.99 (0.62–1.58)	
Adrenal metastasis	Negative	Referent	0.0035	Referent	0.021
	Positive	2.06 (1.27–3.34)		1.90 (1.10–3.27)	
Previous treatment	ICI-pretreated	Referent	0.91	Referent	0.83
	ICI-untreated	1.04 (0.51–2.15)		0.97 (0.75–1.26)	
		**Overall Survival**			
		**Univariate Analysis**		**Multivariate Analysis**	
**Factors**		**Hazard Ratio**	***p*** **Value**	**Hazard Ratio**	***p*** **Value**
Age	<70	Referent	0.15	Referent	0.036
	>=70	1.33 (0.9–1.95)		1.56 (1.03–2.36)	
Gender	Male	Referent	0.44	Referent	0.79
	Female	0.81 (0.47–1.38)		0.93 (0.52–1.64)	
Performance status *	0–2	Referent	0.000043	Referent	0.000025
	3	59.87 (8.43–425.1)		77.60 (10.26–587.10)	
Brain metastasis	Negative	Referent	0.19	Referent	0.17
	Positive	1.30 (0.87–1.93)		1.34 (0.88–2.04)	
Liver metastasis	Negative	Referent	0.0021	Referent	0.081
	Positive	1.91 (1.27–2.89)		1.52 (0.95–2.43)	
M. pleural effusion	Negative	Referent	0.00085	Referent	0.00035
	Positive	2.11 (1.36–3.28)		2.35 (1.47–3.75)	
Bone metastasis	Negative	Referent	0.42	Referent	0.38
	Positive	1.19 (0.79–1.79)		1.23 (0.78–1.95)	
Adrenal metastasis	Negative	Referent	0.0056	Referent	0.021
	Positive	1.95 (1.22–3.13)		1.84 (1.10–3.09)	
Previous treatment	ICI-pretreated	Referent	0.51	Referent	0.46
	ICI-untreated	1.10 (0.83–1.45)		1.11 (0.83–1.49)	

* By the Eastern Cooperative Oncology Group (ECOG). ICI: immune checkpoint inhibitor. M: malignant.

**Table 4:** Hazard ratio adjusted by propensity score.

		n	**Time-to-Treatment Failure**	
			**Hazard Ratio (95% CI)**	***p*-Value**
**unadjusted**	**ICI-pretreated**	27	Referent	0.26
	ICI-untreated	96	1.14 (0.9–1.44)	
IPTW weighted	ICI-pretreated	27	Referent	0.15
	ICI-untreated	96	1.31 (0.91–1.89)	
1:1 matching	ICI-pretreated	21	Referent	0.71
	ICI-untreated	22	1.13 (0.61–2.09)	
			Progression-free survival	
			Hazard ratio (95% CI)	*p*-value
unadjusted	ICI-pretreated	27	Referent	0.91
	ICI-untreated	96	1.04 (0.51–2.15)	
IPTW weighted	ICI-pretreated	27	Referent	0.76
	ICI-untreated	96	0.94 (0.64–1.39)	
1:1 matching	ICI-pretreated	21	Referent	0.88
	ICI-untreated	22	1.05 (0.55–2)	
			Overall survival	
			Hazard ratio (95% CI)	*p*-value
unadjusted	ICI-pretreated	27	Referent	0.51
	ICI-untreated	96	1.10 (0.83–1.45)	
IPTW weighted	ICI-pretreated	27	Referent	0.18
	ICI-untreated	96	1.65 (0.8–3.42)	
1:1 matching	ICI-pretreated	21	Referent	0.48
	ICI-untreated	22	1.29 (0.64–2.6)	

CI: confidence intervals; ICI: immune checkpoint inhibitor; IPTW: inverse probability of treatment weighting.

**Table 5:** Adverse events *.

	ICI-Pretreated Group		ICI-Untreated Group	
N	27		96	
	Any Grade	Grade 3 <=	Any Grade	Grade 3 <=
Febrile neutropenia	6 (22.2)	6 (22.2)	23 (24.0)	23 (24.0)
Anemia	23 (85.2)	3 (11.1)	68 (70.8)	13 (13.5)
Neutropenia	23 (85.2)	17 (63.0)	87 (90.6)	68 (70.8)
Thrombocytopenia	16 (59.3)	5 (18.5)	50 (52.1)	18 (18.8)
Pneumonitis	1 (3.7)	1 (3.7)	7 (7.3)	4 (4.2)

* Common Terminology Criteria for Adverse Events version 5.0; ICI: immune checkpoint inhibitor.

## Data Availability

All data are available following a reasonable request from the corresponding author.
